# Community screening for dementia among older adults in China: a machine learning-based strategy

**DOI:** 10.1186/s12889-024-18692-7

**Published:** 2024-05-01

**Authors:** Yan Zhang, Jian Xu, Chi Zhang, Xu Zhang, Xueli Yuan, Wenqing Ni, Hongmin Zhang, Yijin Zheng, Zhiguang Zhao

**Affiliations:** 1 Department of Elderly Health Management, Shenzhen Center for Chronic Disease Control, No.2021, Buxin Road, Shenzhen, Guangdong 518020 China; 2 Shenzhen Yiwei Technology Company, Shenzhen, Guangdong 518000 China; 3 National Engineering Laboratory of Big Data System Computing Technology, Shenzhen University, Shenzhen, Guangdong 518060 China

**Keywords:** AD8, Cognitive impairment, Dementia, Machine learning

## Abstract

**Background:**

Dementia is a leading cause of disability in people older than 65 years worldwide. However, diagnosing dementia in its earliest symptomatic stages remains challenging. This study combined specific questions from the AD8 scale with comprehensive health-related characteristics, and used machine learning (ML) to construct diagnostic models of cognitive impairment (CI).

**Methods:**

The study was based on the Shenzhen Healthy Ageing Research (SHARE) project, and we recruited 823 participants aged 65 years and older, who completed a comprehensive health assessment and cognitive function assessments. Permutation importance was used to select features. Five ML models using BalanceCascade were applied to predict CI: a support vector machine (SVM), multilayer perceptron (MLP), AdaBoost, gradient boosting decision tree (GBDT), and logistic regression (LR). An AD8 score ≥ 2 was used to define CI as a baseline. SHapley Additive exPlanations (SHAP) values were used to interpret the results of ML models.

**Results:**

The first and sixth items of AD8, platelets, waist circumference, body mass index, carcinoembryonic antigens, age, serum uric acid, white blood cells, abnormal electrocardiogram, heart rate, and sex were selected as predictive features. Compared to the baseline (AUC = 0.65), the MLP showed the highest performance (AUC: 0.83 ± 0.04), followed by AdaBoost (AUC: 0.80 ± 0.04), SVM (AUC: 0.78 ± 0.04), GBDT (0.76 ± 0.04). Furthermore, the accuracy, sensitivity and specificity of four ML models were higher than the baseline. SHAP summary plots based on MLP showed the most influential feature on model decision for positive CI prediction was female sex, followed by older age and lower waist circumference.

**Conclusions:**

The diagnostic models of CI applying ML, especially the MLP, were substantially more effective than the traditional AD8 scale with a score of ≥ 2 points. Our findings may provide new ideas for community dementia screening and to promote such screening while minimizing medical and health resources.

**Supplementary Information:**

The online version contains supplementary material available at 10.1186/s12889-024-18692-7.

## Introduction

Dementia is a leading cause of disability in people older than 65 years worldwide, including China [1]. It is estimated that about 47 million people are currently affected by dementia, and this number is expected to reach 131 million by 2050 [2]. The main clinical manifestation of dementia is significant cognitive decline in one or more cognitive domains that seriously affect the daily lives of patients [3]. The underlying pathology, including amyloid plaque deposition and neurofibrillary tangles, can occur before symptoms appear [2]. Therefore, timely screening, intervention, and treatment for dementia are particularly important.

However, diagnosing dementia in its earliest symptomatic stages remains challenging [4]. The expansion of clinical, epidemiological, and social behavior research is also hampered by the lack of valid screening instruments that can be applied in community settings [5]. Currently, assessments of cognitive function are the most common method of screening for dementia [6]. The Mini-Mental State Examination (MMSE) is the most widely used assessment tool by frontline physicians. The test assesses a wide range of cognitive abilities, such as orientation, memory, arithmetic, language use and comprehension, and basic motor skills [7]. Informant-based assessments provide the opportunity to collect the measurement results of changes and interference levels, but their accuracy depends on the assessed individual’s age and education level, which can be time-consuming and impractical for large-scale community screening, epidemiological field investigations, and locations outside professional centers [5]. A brief informant questionnaire, AD8, was developed at Washington University to detect dementia. The AD8 consists of eight yes–no questions, and a score ≥ 2 suggests cognitive impairment (CI). The AD8 takes less than 3 min to complete and is effective regardless of language, education, culture, or race, making it an apt preliminary screening tool for dementia [8, 9].

It is worth considering that previous studies on AD8 were conducted in settings with an abnormally high prevalence of dementia. But in community settings, the prevalence of dementia may be much lower, such that the effectiveness of AD8, such as positive predictive values, would be correspondingly reduced [10]. Therefore, we speculate that simply using a total score of ≥ 2 as a criterion for community dementia screening may overlook the difference in the weight of eight individual questions. In addition, demographics, lifestyle, and the health-related characteristics of older adults are widely known to be related to CI [11]. Although these characteristics are often collected during daily physical examinations or medical processes in older population, they are generally studied as risk factors and are rarely used to screen for dementia.

In consideration of the lack of simple and efficient dementia screening tools in community settings, this study is based on older adults in China and combines the eight questions of the AD8 scale with comprehensive health-related characteristics. Machine learning (ML) is used to construct diagnostic models of CI. We aimed to provide new ideas and methodological references on how to fully utilize AD8 items (rather than simply using score ≥ 2) and easily accessible health parameters to improve the efficiency of dementia screening among older adults in the communities while minimizing medical and health resources.

## Methods

### Study design and population

The study was based on the Shenzhen Healthy Ageing Research (SHARE) project, which recruited participants aged 65 years and older who had attended the Older Adult Health Management Project of the National Basic Public Health Service in Shenzhen since 2018 [12]. New recruitment and follow-up surveys take place every year. During the fifth year of SHARE (2022), we adopted a multi-stage random sampling method to select subjects for inclusion in this study. First, based on a geographical distribution, we selected a certain number of community health service institutions from 10 administrative districts in Shenzhen city, for a total of 13 selected investigation points. Then, eligible seniors were randomly recruited from each investigation point as participants of this study. Older individuals who were conscious were included. Those diagnosed with Alzheimer’s disease or a disability causing them to be bedridden or unable to communicate adequately, and those unwilling to be investigated were excluded.

From January 1st to December 31st, 2022, we conducted a comprehensive health assessment on older participants as a follow-up survey of SHARE. At the same time, additional cognitive function assessments were performed. A total of 906 older individuals were recruited for this study, and 823 participants who completed all examinations with complete information were included in the analysis, resulting in an effective response rate of 90.84%.

### Comprehensive health assessment

Detailed items and data collection methods of comprehensive health assessment have been described in previous publications [12, 13]. In brief, sociodemographic characteristics, lifestyle, and health-related parameters were collected by a structured questionnaire [13], including sex (male, female), age, educational level (illiteracy, primary school, junior high school and above), marital status (unmarried, divorced, widowed, married), occupation, drinking status (never, occasionally, often), smoking status (never a smoker, ex-smoker, current smoker), exercise (no exercise, occasional exercise, regular exercise), self-assessment of health status (unsatisfactory, satisfactory), self-care ability (good, poor), emotional status screening (negative, positive), and the total scores of the AD8 scale and category of each question, namely A1–A8 (negative, positive). A detailed physical examination was performed to collect information on the participants, including respiratory rate, visual condition, body height, weight, waist circumference (WC), systolic blood pressure and diastolic blood pressure [14]. Body mass index (BMI) was calculated by dividing the participants’ body weight by the square of their height. Electrocardiography measurements were taken to measure the heart rate and check for heart abnormalities [12]. A fasting blood sample of the participants was collected to obtain information on the levels of hemoglobin (HB), white blood cells (WBCs), platelets, serum uric acid, serum creatinine, alanine aminotransferase, glutamic oxalacetic minotransferase, total bilirubin, total cholesterol, triglycerides, low-density lipoprotein cholesterol, high-density lipoprotein cholesterol, fasting plasma glucose and carcinoembryonic antigen [15]. According to our previous studies, several chronic diseases were defined, including hypertension, diabetes, dyslipidemia, anemia, chronic kidney disease (CKD), and liver dysfunction [14–16].

### Cognitive function assessment

All participants underwent a cognitive assessment (dementia screening) using the Chinese version of the MMSE, which was valid and reliable for screening Chinese after taking cultural and linguistic differences into account [17]. The test was conducted following guidelines and protocols by trained investigators and typically took 15–20 min to complete. The sum of all item points produced total scores, ranging from 0 to 30. A higher score indicates better cognitive function [18]. CI was identified using education-specific cutoff points of the total MMSE score, as follows: no formal education, 17/18; elementary education, 20/21; and middle school or greater education, 24/25 [19]. All data were collected by investigators specially trained for this study, including doctors and nurses.

### Statistical analysis

All participants were divided into a CI group and a cognitively normal (CN) group. We began with a descriptive analysis of two group of participants, with the mean ± standard deviation (SD) and median (interquartile range, IQR) for quantitative variables, frequencies and proportions for categorical variables. The chi-squared test, t-test, and Mann–Whitney test were used to compare the sociodemographics, lifestyle characteristics, and health-related parameters between the two groups. Differences were found to be statistically significant using two-tailed significance tests (*P* ≤ 0.05). All statistical analyses were performed using SPSS software (IBM SPSS Statistics 25.0, IBM Corporation).

### Predictive modeling pipeline

**Fig. 1:**
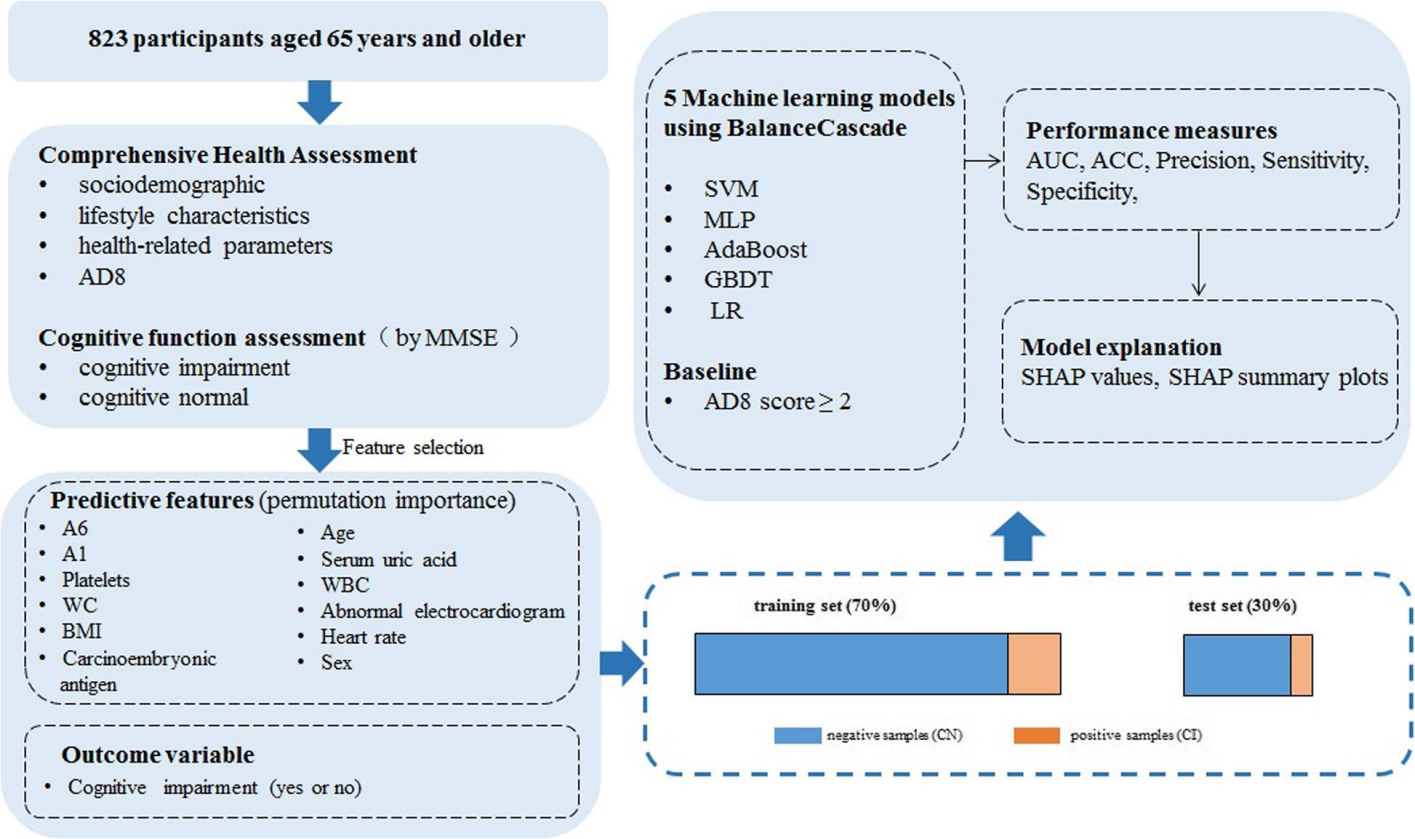
The processes of building CI diagnostic models (CI: cognitive impairment)

To build effective diagnostic models of CI in older adults, ML and related processes were carried out, including data preprocessing, feature selection, ML processing, performance measures and model explanation, as shown in Fig. 1. Comprehensive health assessment variables were used as predictive features, and CI (yes or no) was used as outcome variable.

#### Data preprocessing

Samples with missing values and excessively abnormal feature values based on professional judgment were excluded to reduce noisy training instances. A total of 823 participants were classified as CI group and CN group, representing positive and negative samples, respectively. Categorical features such as educational level, marital status, etc. were one-hot encoded into separate features. For scale-sensitive models such as multilayer perceptron (MLP), support vector machine (SVM), and logistic regression (LR), standard scaling was conducted to eliminate scale differences. The preprocessed dataset included 823 samples and 54 features.

#### Feature selection

We used permutation importance to select features. This technique measures the contribution of each feature to the model by observing the resulting degradation of the model’s score when a specific feature value was randomly shuffled [20]. The relative importance was calculated for each feature. Those features with a mean importance greater than twice their standard deviation were included subsequently in the ML models.

#### Machine learning processing

Our ML models were presented to solve a binary classification problem. We started with two known classes (CI and CN), and we sought to obtain the model that best differentiated these classes and classified individuals to determine whether a subject belonged to a specific class. Firstly, maintaining the original distribution of two classes, the data was randomly divided into two-thirds as the training set and the remaining one-third as the test set. Then, in the training set, we implemented five ML algorithms to build diagnostic models, including SVM, MLP, AdaBoost, gradient boosting decision tree (GBDT), and LR. Considering the data sets were imbalanced in the CI and CN classes (approximately 1:10), we used BalanceCascade, an ensemble strategy to train models. BalanceCascade sample multiple subsets of the majority class, train an ensemble from each of these subsets, and combine all weak classifiers in these ensembles into a final output. Unlike other ensemble strategies, BalanceCascade trains the learners sequentially, where in each step the majority class examples which are correctly classified by the current trained learners are removed from further consideration [21]. We also implemented single model training (non-ensemble strategy) as an additional reference.

#### Performance measures

After the models were built, they were scored and evaluated using the test-set data. The performance of the models was measured using the area under the curve (AUC), accuracy (ACC), sensitivity (Sen), and specificity (Spe). Receiver operating characteristic (ROC) curves were drawn to show the recognition capability of the models. At the same time, we used parameters of the AD8 score ≥ 2 to define cognitive impairment as a baseline for comparing the performance of the ML models.

#### Model explanation

SHapley Additive exPlanations (SHAP) values were used to help interpret the results of the ML models. SHAP summary plots of the models for predicting CI were drawn. All plots illustrate the SHAP value changes when the values of a feature increase or decrease, showing the direction and degree of influence on the model’s decision through the SHAP value of each feature [22].

#### Software

The experimental codes were implemented using Python 3. Feature selection and the standardization of features and ML algorithms (SVM, MLP, AdaBoost, GBDT, and LR) were implemented using the “Scikit-learn library”, and for SHAP using the “shap” library.

## Results

### Characteristics of the study population

Of the 823 older participants, 72 (8.75%) were assessed as having CI, and 751 (91.25%) were CN according to the MMSE. The differences in sociodemographics, lifestyle characteristics, and health-related parameters between the CN and CI groups are described in Table 1. In terms of demographics, the median age of the CI group (72.5 years) was older than that of the CN group (71 years). For lifestyle characteristics, the CN group had a higher proportion of regular exercise. In terms of health-related parameters, the average BMI and the WC of the CI group were lower. In addition, the proportions of unsatisfactory self-assessment of health status, poor self-care ability, positive emotional status screening, abnormal electrocardiogram, anemia, CKD, AD8 scores ≥ 2, and each of eight positive items in the CI group were higher than in the CN group.

**Table 1:** Characteristics of the subjects in CN and CI groups

Characteristics	CN (*n* = 751)	CI (*n* = 72)	*P* values
**Sociodemographic characteristics**			
Sex-male, n (%)	337 (44.9)	26 (36.1)	0.153
Age (median [IQR]) *	71 (7)	72.5 (12)	0.007
Educational level- junior high school and above, n (%)	579 (77.1)	49 (68.1)	0.051
Marital status- married, n (%)	724 (96.4)	67 (93.1)	0.16
No occupation, n (%)	158 (21.0)	24 (33.3)	0.283
**Lifestyle characteristics**			
Regular exercise, n (%) *	623 (83.0)	52 (72.2)	0.023
Current smoker, n (%)	58 (7.7)	5 (6.9)	0.972
Often drinking, n (%)	50 (6.7)	4 (5.6)	0.476
**Health-related parameters**			
BMI (mean (SD)) *	24.13 (3.1)	22.89 (3.7)	0.001
WC (median [IQR]) *	85 (12)	82.5 (10.8)	0.008
Heart rate (median [IQR])	72 (13)	72 (12)	0.924
Respiratory rate (median [IQR])	18 (1)	18 (1)	0.228
WBC (median [IQR])	5.94 (1.9)	5.95 (2.2)	0.619
Platelets (median [IQR])	215 (76)	202 (94.3)	0.702
Carcinoembryonic antigen (median [IQR])	1.99 (1.5)	2.26 (1.6)	0.099
Serum uric acid (median [IQR])	341 (116)	334.5 (135.6)	0.887
Unsatisfactory self-assessment of health status n (%) *	54 (7.2)	10 (13.9)	0.043
Poor self-care ability, n (%) *	4 (0.5)	2 (2.8)	0.032
Positive emotional status screening, n (%) *	55 (7.3)	15 (20.8)	< 0.001
Visual impairment, n (%)	80 (10.7)	8 (11.1)	0.904
Abnormal electrocardiogram, n (%) *	381 (50.7)	47 (65.3)	0.018
Anemia, n (%) *	83 (11.1)	14 (19.4)	0.035
Liver dysfunction, n (%)	197 (26.2)	20 (27.8)	0.776
CKD, n (%) *	37 (4.9)	11 (15.3)	< 0.001
Hypertension, n (%)	435 (57.9)	50 (69.4)	0.058
Diabetes, n (%)	185 (24.6)	22 (30.6)	0.269
Dyslipidemia, n (%)	365 (48.6)	32 (44.4)	0.5
AD8 score ≥ 2, n (%) *	238 (31.7)	39 (54.2)	< 0.001
A1-positive, n (%) *	39 (5.2)	15 (20.8)	< 0.001
A2-positive, n (%) *	60 (8.0)	16 (22.2)	< 0.001
A3-positive, n (%) *	70 (9.3)	11 (15.3)	0.105
A4-positive, n (%) *	32 (4.3)	12 (16.7)	< 0.001
A5-positive, n (%) *	38 (5.1)	14 (19.4)	< 0.001
A6-positive, n (%) *	48 (6.4)	20 (27.8)	< 0.001
A7-positive, n (%) *	114 (15.2)	26 (36.1)	< 0.001
A8-positive, n (%) *	311 (41.4)	44 (61.1)	0.001

*CN* Cognitively normal, *CI* Cognitive impairment, *BMI* Body mass index, *WC* Waist circumference, *WBC* White blood cell, *CKD* Chronic kidney disease, *SD* Standard deviation, *IQR* Interquartile range

*Represented significant statistical differences

### Feature importance

**Fig. 2:**
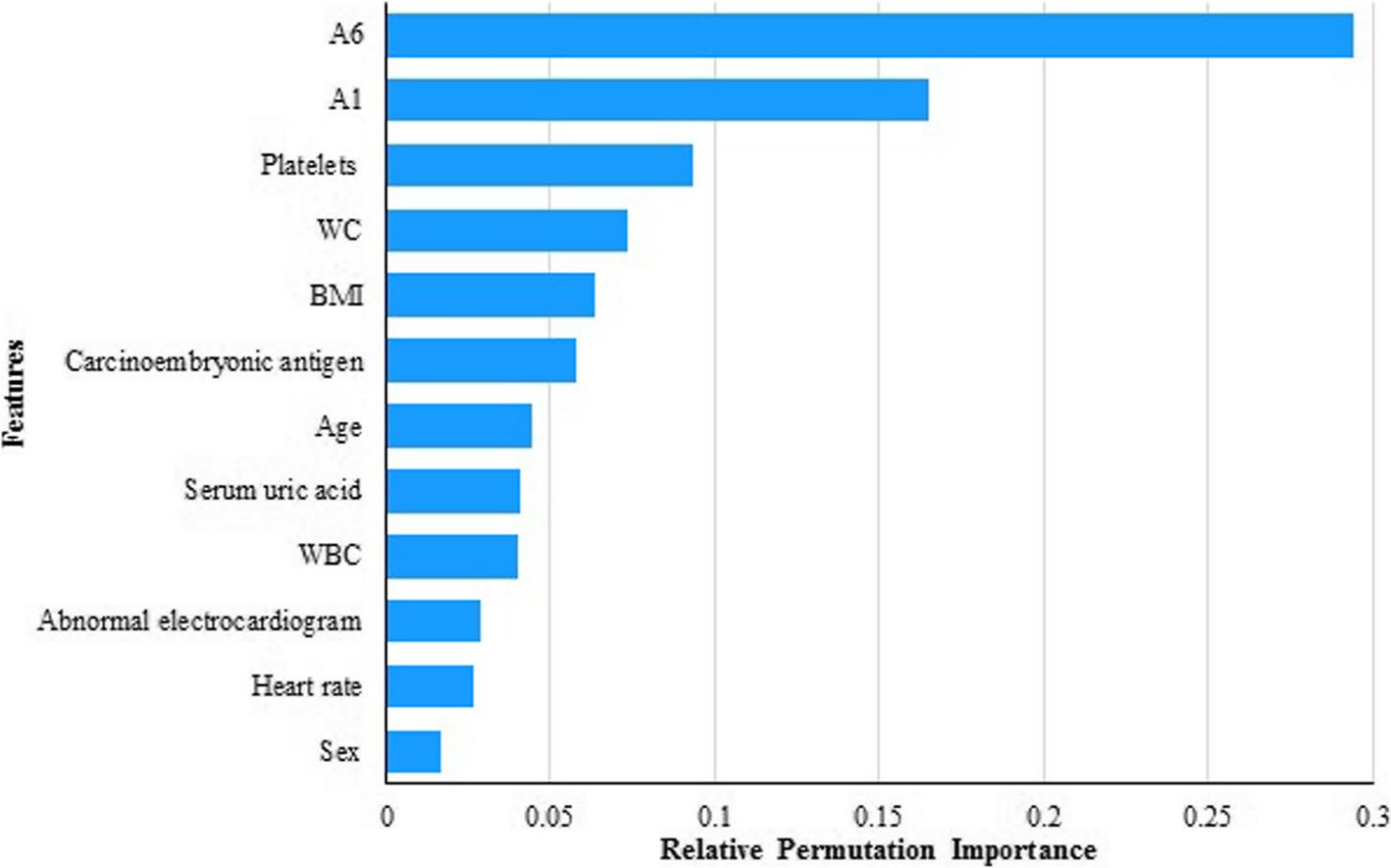
The permutation importance of selected features

According to the results of permutation importance, the top five features with the greatest importance for predicting CI are the sixth item of AD8 (A6), first item of AD8 (A1), platelets, WC, and BMI, followed by carcinoembryonic antigen, age, serum uric acid, WBC, abnormal electrocardiogram, heart rate, and sex (Fig. 2). These features were used as predictive features for subsequent ML.

### Model performance

**Fig. 3:**
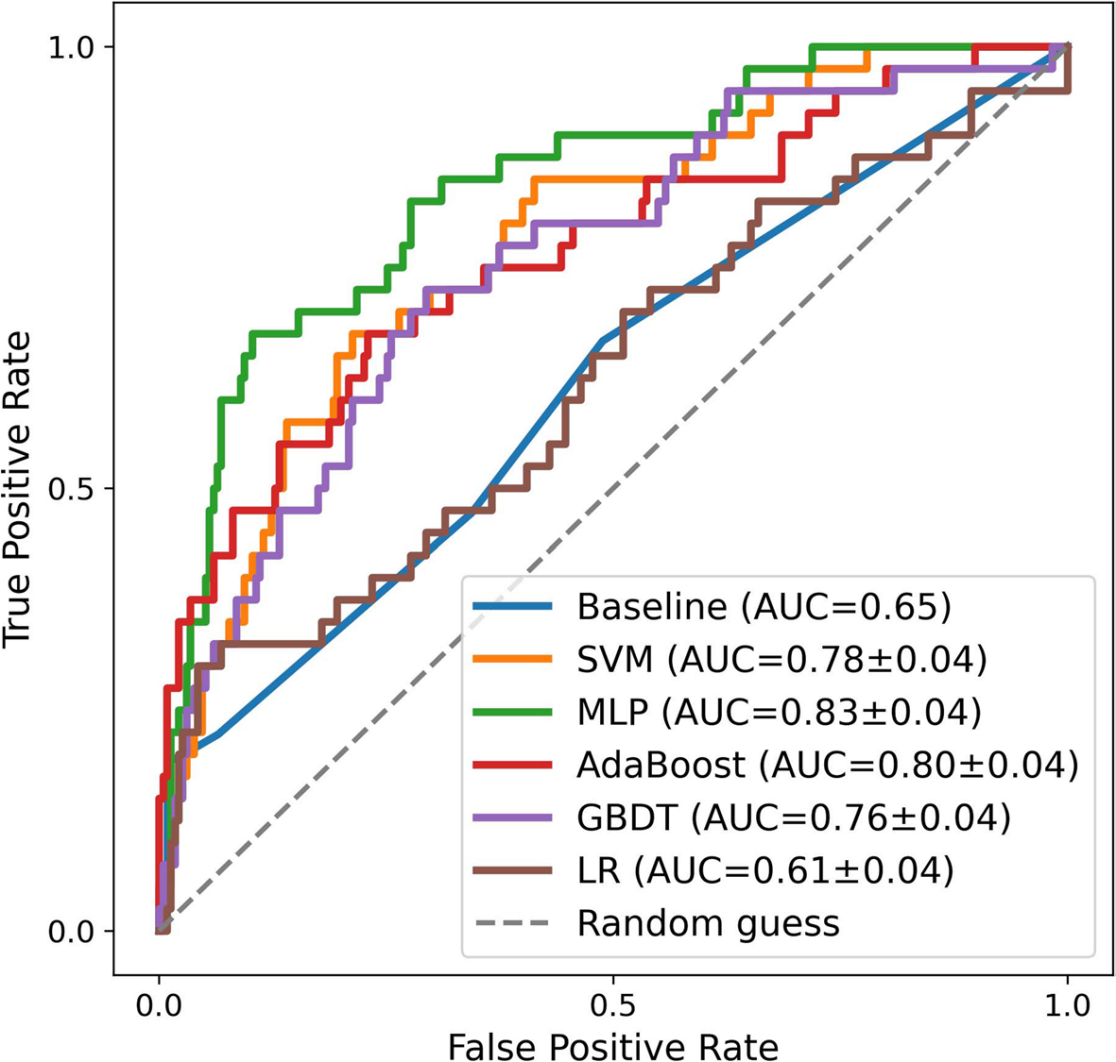
ROC curve of five ML models and baseline for CI prediction (ML: machine learning; CI: cognitive impairment)

Regarding the models’ effectiveness at predicting CI, compared to the baseline, which evaluates CI with an AD8 score of ≥ 2 (AUC = 0.65), all four ML models except the LR showed better performance overall. The MLP showed the highest performance (AUC: 0.83 ± 0.04), followed by AdaBoost (AUC: 0.80 ± 0.04), SVM (AUC: 0.78 ± 0.04), and GBDT (0.76 ± 0.04). Furthermore, the accuracy, sensitivity and specificity of four ML models were higher than the baseline (Table 2), which indicated that these models have a better ability to correctly classify positive and negative samples than the baseline. Figure 3 illustrates the CI predictions for the algorithm at each optimum. The ROC curves for each prediction model are represented by different colored lines. The results of these models trained using non-ensemble strategy were presented in the Supplementary material 1.

**Table 2:** Performance comparison between five models and baseline method

**Models**	**ACC (mean ± SD)**	**Precision (mean ± SD)**	**Sen (mean ± SD)**	**Spe (mean ± SD)**	**AUC (mean ± SD)**
Baseline (Ad8 ≥ 2)	0.36	0.54	0.53	0.75	0.65
SVM	0.77 ± 0.07	0.47 ± 0.05	0.66 ± 0.12	0.79 ± 0.10	0.78 ± 0.04
MLP	0.83 ± 0.05	0.55 ± 0.06	0.70 ± 0.09	0.85 ± 0.07	0.83 ± 0.04
AdaBoost	0.79 ± 0.06	0.48 ± 0.05	0.67 ± 0.11	0.81 ± 0.09	0.80 ± 0.04
GBDT	0.76 ± 0.07	0.44 ± 0.05	0.63 ± 0.13	0.78 ± 0.10	0.76 ± 0.04
LR	0.70 ± 0.15	0.31 ± 0.04	0.46 ± 0.22	0.74 ± 0.21	0.61 ± 0.04

*ACC* Accuracy, *Sen* Sensitivity, *Spe* Specificity, *AUC* Area under the curve, *SVM* Support vector machine, *MLP* Multilayer perceptron, *GBDT* Gradient boosting decision tree, *LR* Logistic regression

### Model explanation

**Fig. 4:**
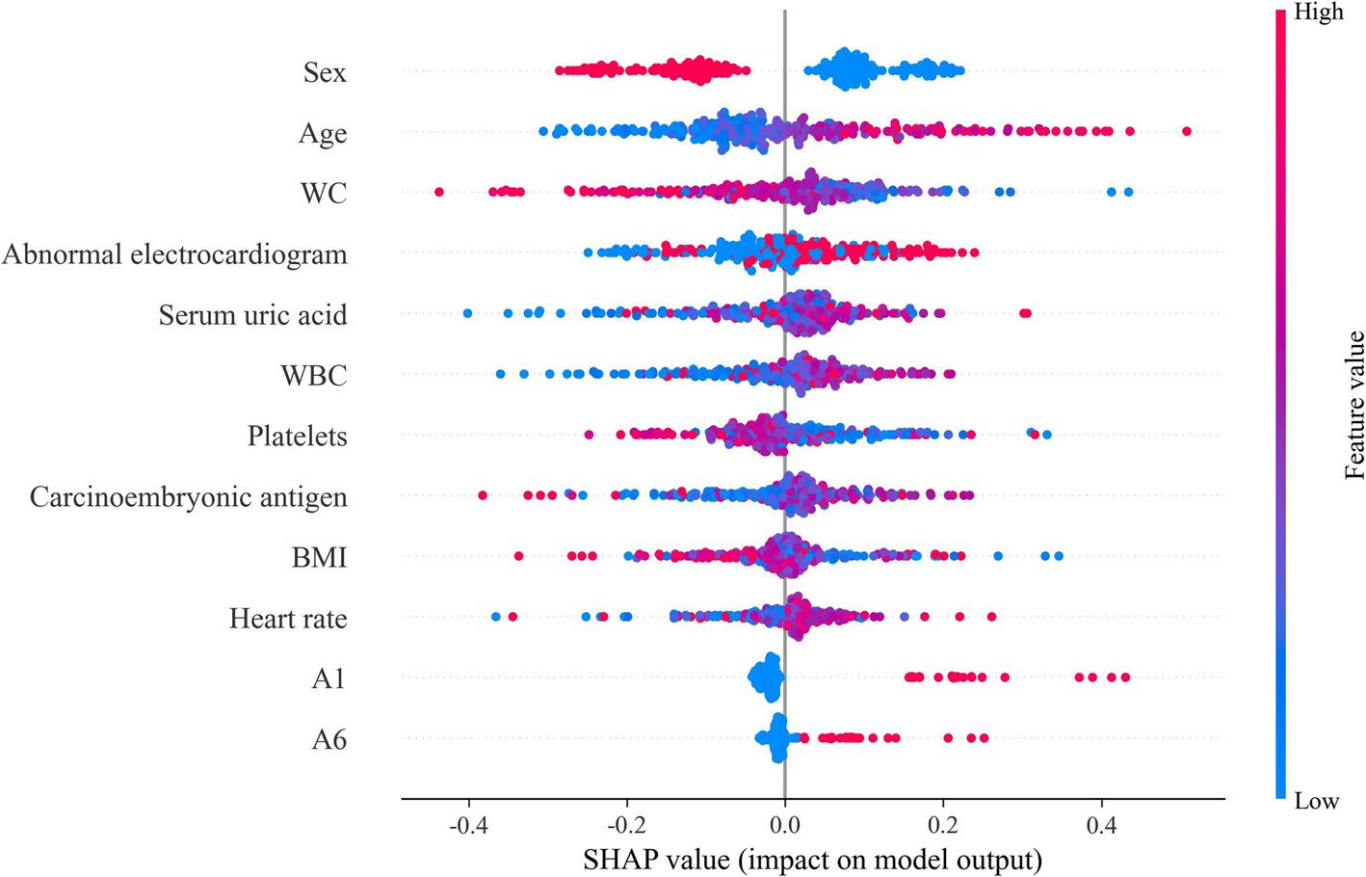
SHAP summary plots for CI prediction based on MLP (CI: cognitive impairment; MLP: multilayer perceptron): The horizontal coordinates of the sample points indicate their SHAP values and order of features along the vertical axis based on the sum of SHAP values of all samples. The vertical coordinates are determined by the feature where the point is located, and the colors of the points, ranging from blue to red, represent the sample feature values from small to large. Red dots with positive SHAP value and blue dots with negative SHAP value mean that a higher value promotes CI occurrence and a lower value hinders CI occurrence

SHAP summary plots were based on the MLP and illustrated how each feature affect the model’s judgment of CI. As shown in Fig. 4, the most influential feature on model decision for positive CI prediction was provided by female sex, followed by older age and lower WC. Furthermore, higher abnormal electrocardiogram, serum uric acid, WBC, carcinoembryonic antigen and heart rate, lower platelets level and BMI, positive A1 and A6 items also increased the risk of CI.

## Discussion

In this study, we applied ML to build diagnostic models of CI among older adults in China. In particular, through a feature selection process, we used some specific items in AD8 (a brief dementia screening scale), together with sociodemographics, lifestyle characteristics, and health-related parameters of the older adults, as the predictive features of CI. To the best of our knowledge, this is the first study to compare the effectiveness of ML with traditional brief scales. We observed better power for identifying CI from ML models (especially the MLP) than traditional AD8 scale. Therefore, our research process could be applied to identify older individuals who are more likely to have CI, when completing the two items of AD8 (A1 and A6), and obtaining a few easily accessible health parameters, which provided a new perspective for community screening for dementia without conducting complex cognitive function assessment scales.

In our study, the prevalence of CI among older adults in a representative region of China was 8.75%, which is lower than the observed values in other studies [23, 24]. This is normal because the results are influenced by the evaluation method and the population composition of different regions. But it is worth affirming that the marked decline in the utility of the AD8 may be expected in settings with dementia prevalence rates more in line with community-based estimates [10]. This underscores the importance of choosing tools with optimal characteristics when screening communities for dementia.

The distribution difference of some sociodemographic features, lifestyle characteristics, and health-related parameters between the CI group and CN group indicated the availability of these factors in predicting CI. In order to better utilize these characteristics, as well as individual items from the AD8 scale—rather than simply using the total score—we screened several important features based on their importance and used ML to construct diagnostic models for CI. In recent years, ML algorithms have been used to detect a variety of diseases [25–27], and were developed to analyze large, complex datasets in medical settings and clinical environments [28]. Indeed, ML is believed to optimize the prediction of CI and overcome the shortcomings of traditional methods [29]. Although some studies have reported the usefulness of ML to predict patients with CI [29–31], few have compared the effectiveness of ML with traditional scales, especially tools such as AD8 that are widely used in community screening. By doing so, we can facilitate the development of more efficient community dementia-screening methods or tools.

We used five ML models: SVM, MLP, AdaBoost, GBDT, and LR. These models are frequently used for classification [26, 29, 32–34]. We used the traditional dementia screening method, AD8, with a score of ≥ 2 as the baseline. Four ML models demonstrated better performance at CI prediction. Among them, the MLP exhibited the best predictive ability, with a higher AUC (0.83 ± 0.04) than some previous studies about CI prediction [31, 35], and has  a long history of implementation in medical research for classification, detection, and prediction [32]. It is worth emphasizing that an ensemble training method based on BalanceCascade adopted to handle imbalanced data sets may have certain reference value for some research related to ML. After all, class-imbalance is a common phenomenon in medical research related to disease diagnosis [33, 36].

Moreover, SHAP values were used to explain the MLP classification results and reveal the significance of the considered factors. According to the SHAP values, being female and older age were important features for predicting positive CI. This is consistent with previous research results [23, 37]. In terms of other health-related characteristics, lower WC, platelets level and BMI, higher abnormal electrocardiogram, serum uric acid, WBC, carcinoembryonic antigen and heart rate contributed significantly to CI. This is also consistent with past discoveries [24, 38–40]. These factors have been associated with CI, but they are rarely used together to predict CI. Specifically, a positive value for the first AD8 question (A1) and the sixth question (A6) contributed to the prediction of CI. A1 and A6 represent that the subject has judgment problems and economic transaction processing difficulties, respectively. Indeed, AD8 was designed primarily as a screening tool to identify individuals at risk, for broader staging and differential diagnosis, such as neuropsychological testing [5]. Our findings suggest that some of items of AD8 may be more important than others for predicting CI. However, more research is needed to explore the consistency and contribution weights of each item with more detailed assessments of dementia and gold standards such as biomarkers, in order to strengthen the case for a full utilization of this brief community dementia-screening tool, rather than simply calculating the total score.

## Conclusions

The diagnostic models of CI applying ML, especially the MLP, were substantially more effective than the traditional AD8 scale with a score of ≥ 2 points. Our findings provide new insights on how to use demographics and health parameters in combination with a few important items in the AD8 scale to strengthen dementia screening of the older adults in communities, and they can serve as a reference for targeted intervention of individuals at risk.

## Limitations

This study has some limitations. Firstly, several samples with excessively abnormal feature values were excluded based on professional judgment, which may not conform to standard clinical practice guidelines. In addition, relatively small proportion of patients with CI were included. ML models are more powerful when they consider lots of patients. Finally, the evaluation of patients with CI was based on a commonly used neuropsychology test, and its diagnostic performance for dementia and mild CI was limited. However, our aim was to strengthen community dementia screening, and this can be verified in more clinical dementia patients in the future.

### Supplementary Information

**Supplementary Material 1.**

## Data Availability

The data cannot be made available publicly due to an ethical restriction as the consent of participants implied that only the research team will have access to the data provided for the study. Anonymised data from the study is held by Dr Zhiguang Zhao. Those interested in obtaining the data and study materials should contact Dr Zhiguang Zhao to request appropriate approval for access.

## Abbreviations

ML: Machine learning
CI: Cognitive impairment
SVM: Support vector machine
MLP: Multilayer perceptron
GBDT: Gradient boosting decision tree
LR: Logistic regression
SHAP: SHapley Additive exPlanations
CN: Cognitively normal
BMI: Body mass index
WC: Waist circumference
WBC: White blood cell
CKD: Chronic kidney disease
SD: Standard deviation
IQR: Interquartile range
ACC: Accuracy
Sen: Sensitivity
Spe: Specificity
AUC: Area under the curve
