# Supplementary material for: Community screening for dementia among older adults in China: a machine learning-based strategy
Source: BMC Public Health. 2024 May 1;24:1206. doi: 10.1186/s12889-024-18692-7 (PMC11062005; doi:10.1186/s12889-024-18692-7)
Supplement: Supplementary file 1 — Supplementary Material 1. [file 12889_2024_18692_MOESM1_ESM.docx]

Table. The performance of models trained using ensemble methods vs using single model training

| **Models** | **ACC**  **(mean±SD)** | | **Sen**  **(mean±SD)** | | **Spe**  **(mean±SD)** | |
| --- | --- | --- | --- | --- | --- | --- |
|  | Strategy 1 | Strategy 2 | Strategy 1 | Strategy 2 | Strategy 1 | Strategy 2 |
| SVM | 0.77±0.07 | 0.89±0.01 | 0.66±0.12 | 0.31±0.07 | 0.79±0.10 | 0.90±0.01 |
| MLP | 0.83±0.05 | 0.88±0.03 | 0.70±0.09 | 0.54±0.12 | 0.85±0.07 | 0.93±0.02 |
| AdaBoost | 0.79±0.06 | 0.88±0.01 | 0.67±0.11 | 0.16±0.07 | 0.81±0.09 | 0.88±0.01 |
| GBDT | 0.76±0.07 | 0.87±0.01 | 0.63±0.13 | 0.17±0.08 | 0.78±0.10 | 0.88±0.01 |
| LR | 0.70±0.15 | 0.87±0.01 | 0.46±0.22 | 0.08±0.05 | 0.74±0.21 | 0.87±0.01 |

Strategy 1 represents ensembled models training using BalanceCascade, strategy 2 represents using single model training.
